# Prognostic Evaluation and Functional Characterization of Cyclin K Expression in Endometrial Cancer: Immunohistochemical and In Silico Analysis

**DOI:** 10.3390/cancers17050792

**Published:** 2025-02-25

**Authors:** Marcin Szymański, Klaudia Bonowicz, Dominika Jerka, Maciej Gagat, Paulina Antosik

**Affiliations:** 1 Department of Histology and Embryology, Collegium Medicum in Bydgoszcz, Nicolaus Copernicus University in Torun, 85-092 Bydgoszcz, Poland; md.marcinszymanski@gmail.com (M.S.); klaudia.bonowicz@cm.umk.pl (K.B.); dominika.jerka@cm.umk.pl (D.J.); 2 Faculty of Medicine, Collegium Medicum, Mazovian Academy in Płock, 09-402 Płock, Poland; 3 Department of Clinical Pathomorphology, Faculty of Medicine, Collegium Medicum in Bydgoszcz, Nicolaus Copernicus University in Toruń, 85-094 Bydgoszcz, Poland; paulina.antosik@cm.umk.pl

**Keywords:** CCNK, endometrial cancer, prognostic factor

## Abstract

**Background/Objectives:** Endometrial cancer (EC) is a heterogeneous gynecological malignancy characterized by varied clinical outcomes and complex molecular mechanisms. The dysregulation of cyclin K (CCNK), a key regulator of transcription and cell cycle progression, has been implicated in cancer development. This study aimed to investigate CCNK expression at the protein level in EC tissues and at the mRNA level using in silico analysis. Additionally, the prognostic significance of CCNK expression in EC was assessed. **Methods:** CCNK expression was evaluated using immunohistochemical analysis and mRNA expression profiling in EC tissues, adjacent non-tumorous tissues, and histologically normal endometrial tissues. Immunohistochemical staining was performed on tissue macroarrays, and protein expression was quantified using the Immunoreactivity Score (IRS). mRNA expression analysis was conducted in silico using TCGA data via UCSC Xena and UALCAN web tool. Pathway enrichment was analyzed using Reactome and DAVID tool, while PPI networks were constructed with STRING and Cytoscape. Statistical analyses, including Mann–Whitney U test, Fisher’s exact test, Chi-square test, Kaplan–Meier survival analysis, and Cox regression, were performed using GraphPad Prism. **Results:** Immunohistochemical analysis revealed significantly elevated CCNK protein expression in tumor tissues, particularly in advanced-stage cases, correlating with adverse pathological features such as higher tumor stage and FIGO grade. High CCNK protein expression was significantly associated with poorer OS in the overall EC cohort and non-endometrioid subtypes, whereas no significant association was observed in endometrioid subtypes. mRNA expression analysis demonstrated significantly higher *CCNK* levels in non-endometrioid tumors compared to adjacent non-tumorous tissues, but no significant correlation with OS was observed. Functional enrichment analysis highlighted the involvement of *CCNK*-associated genes in RNA metabolism and transcriptional regulation. **Conclusions:** These findings emphasize the prognostic value of CCNK expression in EC, particularly in aggressive subtypes. The results suggest that CCNK may serve as a potential therapeutic target, warranting further investigation into its role in EC progression and treatment strategies.

## Simple Summary

This study examines the expression of cyclin K (CCNK) in endometrial cancer by comparing immunohistochemical analysis (IHC) with gene expression data from The Cancer Genome Atlas (TCGA) across endometrioid and non-endometrioid types and evaluates its correlation with patient survival outcomes. Our findings indicate that CCNK expression is elevated in endometrial cancer tissues compared to normal endometrial samples. Higher protein and mRNA expression levels were associated with worse overall survival (OS). A functional enrichment analysis of genes linked to *CCNK* suggests its involvement in transcriptional regulation and RNA metabolism. These results contribute to understanding the potential role of CCNK in tumor biology and its prognostic significance in endometrial cancer.

## 1. Introduction

EC constitutes 90% of all uterine cancer cases, making it one of the most prevalent gynecological malignancies and a leading cause of cancer among women globally [1]. The majority of patients with endometrial cancer are diagnosed at an early stage, where standard treatment typically involves surgical intervention, possibly combined with adjuvant radiotherapy or chemotherapy based on the assessed risk of disease recurrence [2]. EC is associated with hyperestrogenism-related factors such as early menarche, late menopause, polycystic ovary syndrome (PCOS), infertility, obesity, and diabetes. Estrogens stimulate endometrial growth and proliferation, playing a key role in EC development. Additionally, genetic predisposition contributes to EC risk, with Lynch syndrome being a notable factor in hereditary cases [3]. Significant progress has been made in improving the management of endometrial cancer, including in early-stage cases. Key advancements such as sentinel lymph node mapping, which detects low-volume metastatic disease, molecular profiling based on The Cancer Genome Atlas (TGCA) framework, and an updated staging system have refined prognostic assessments, paving the way for more personalized treatment strategies [4]. Despite medical advancements, 15% of patients are diagnosed at advanced stages of endometrial cancer. In these stages, specifically International Federation of Gynecology and Obstetrics (FIGO) stages III and IVA-B, the five-year OS rates significantly decline to 40–65% for stage III and 15–17% for stages IVA-B, respectively. Until recently, women with recurrent or metastatic disease (FIGO stage IVC) faced limited therapeutic options, primarily restricted to chemotherapy, which often diminishes in effectiveness after the first line of treatment [5,6].

CDKs play key roles in cancer progression, making them important targets for cancer therapy, as evidenced by several approved CDK4/6 inhibitors in breast and lung cancer and ongoing clinical trials in other tumor types [7]. CDKs are essential for driving cell cycle progression, requiring interaction with regulatory cyclin subunits to achieve catalytic activity [8].

Cyclin K plays a pivotal role in cell division, DNA replication, and genomic stability, supporting cancer cell proliferation and therapy resistance [9,10,11]. Encoded by the CCNK gene, it belongs to the transcription cyclin family and regulates gene expression [12]. Its expression is consistently detected in various cancers, indicating its potential as a biomarker. Cyclin K loss in prostate cancer drives androgen receptor (AR) variant expression and therapy resistance, inducing a BRCA-like deficiency (BRCAness) that increases sensitivity to PARP inhibitors, making it a key target in advanced prostate cancer [13]. Additionally, cyclin K is overexpressed in pancreatic ductal adenocarcinoma (PDAC), correlating with poor survival and promoting G1-S progression via CDC20. Its depletion impairs proliferation in vivo and enhances sensitivity to GemTaxol and PARP inhibitors [14]. In lung cancer, cyclin K stabilizes β-catenin and drives cyclin D1 expression, enhancing tumor proliferation and radioresistance. Silencing cyclin K disrupts the G2/M checkpoint, impairing tumor growth and increasing radiosensitivity, highlighting its therapeutic potential in lung cancer [15]. Cyclin K is increasingly recognized as a key regulator in cancer biology, with studies linking its expression to tumor progression and cell proliferation, underscoring its potential as a therapeutic target [16].

Cyclin K interacts with CDK12 and CDK13 to form active complexes essential for transcription regulation [9]. These complexes are crucial in cell division, and their dysregulation contributes to EC progression [17]. Beyond transcriptional regulation, CDK12 and CDK13 are involved in RNA processing, splicing, and translation control, linking transcription elongation to post-transcriptional modifications [18]. CDK12 ensures proper expression of DNA damage repair genes by preventing premature polyadenylation, while also regulating mitotic genes through direct phosphorylation of translation initiation factors [19]. CDK13 is involved in RNA surveillance, preventing stabilization and translation of aberrant mRNAs, which could contribute to oncogenesis [20]. In BRAF-mutated melanoma, CDK12 activity drives tumor proliferation and genomic stability, while the CDK12/13 inhibitor SR-4835 inhibits DDR gene expression, induces DNA damage, and impairs melanoma growth. SR-4835 also acts as a molecular glue, promoting cyclin K degradation via the CUL4-RBX1-DDB1 ubiquitin ligase complex, with its benzimidazole side-chain being critical for this activity [21].

In this study, we aim to investigate CCNK expression and its potential clinical significance in EC. The initial phase focused on the immunohistochemical evaluation of CCNK expression and distribution in EC tissues and adjacent non-tumor tissues. Protein expression data were analyzed to determine correlations with the clinicopathological features and OS of patients with EC. Additionally, in silico analysis of data from gene expression analyses were performed using datasets from TCGA, comparing *CCNK* expression in endometrial cancer samples with normal endometrial tissue. These analyses aimed to elucidate the transcriptional landscape of *CCNK* and its potential regulatory networks in tumor biology. Functional enrichment and pathway analyses were conducted to identify biological processes, molecular functions, and cellular components associated with *CCNK* expression. By examining gene expression patterns and coexpression networks, we sought to determine potential oncogenic interactions, which may contribute to tumor progression and influence patient prognosis.

## 2. Materials and Methods

### 2.1. Patients and Tissue Material

This study was conducted using archived tissue material from the Department of Clinical Pathomorphology, Collegium Medicum in Bydgoszcz, Nicolaus Copernicus University in Toruń, with ethical approval from the Institutional Ethics Committee (KB/87/2020). The study group comprised 128 patients histologically diagnosed with EC, who underwent abdominal hysterectomy with bilateral salpingo-oophorectomy and lymphadenectomy at the Department of Obstetrics, Gynecological Diseases, and Oncological Gynecology at Dr. Jan Biziel University Hospital No. 2 in Bydgoszcz. The patients ranged in age from 40 to 84 years, with a mean age of 66.2 years and a median age of 66 years. Postoperative histopathological examination confirmed the diagnosis of EC. To account for the heterogeneous nature of the study group and the limited number of cases within specific FIGO classification categories, a simplified classification was applied to reflect trends in disease progression. Key clinical and pathological variables included age (≤60 vs. >60 years), histologic grade (G1, G2, G3), pathological T stage (pT1–pT4), pathological N stage (pN0–pN1), pathological M stage (pM0–pM1), FIGO staging (I–IV), lymphovascular space invasion (LVSI; present vs. absent), and tumor histology (endometrioid vs. non-endometrioid type). The date of 13 April 2022 was set as the cut-off for OS analysis, defined as the time from diagnosis to the last follow-up or death. The median follow-up duration was 106.5 months, during which 58 patients (45.31%) passed away. Postsurgical survival data were available for all patients.

The control group consisted of material obtained from 30 patients who had previously undergone surgical hysterectomy due to uterine fibroids at the same department. The patients’ ages ranged from 45 to 71 years, with a mean age of 60 years. Histopathological examination of the control tissue revealed normal endometrial tissue with no findings associated with proliferation, inflammation, or neoplastic changes.

### 2.2. Immunohistochemistry

Immunohistochemical staining for CCNK was performed on tissue macroarrays constructed from tumor-rich representative areas of paraffin blocks and tumor-adjacent histologically normal tissues. Four-micrometer-thick sections were cut from the macroarrays using a manual rotary microtome (Accu-Cut, Sakura, Torrance, CA, USA) and mounted on adhesion-coated slides (SuperFrost Plus, Menzel-Gläser, Braunschweig, Germany). Sections were dried at 60 °C for 30 min before further processing. The slides were dewaxed in xylene, were rehydrated through graded alcohol concentrations, and underwent antigen retrieval in Ventana high-pH CC1 buffer for 64 min (Roche Diagnostics/Ventana Medical Systems, Tucson, AZ, USA). The sections were incubated with a rabbit monoclonal anti-CCNK antibody (1:50, HPA077073, Sigma Aldrich, St. Louis, MO, USA) for 40 min, and antigen–antibody complexes were visualized using the UltraView Universal DAB Detection Kit (Ventana Medical Systems, Tucson, AZ, USA). Tissue sections were counterstained with hematoxylin for nuclear visualization, dehydrated through graded ethanol concentrations (80%, 90%, 96%, 99.8%), cleared in xylene, and cover-slipped using Dako mounting medium (Agilent Technologies, Santa Clara, CA, USA). Known positive control sections were included, selected based on antibody datasheets and the Human Protein Atlas (http://www.proteinatlas.org, accessed on 12 September 2021), and negative controls were prepared by omitting the primary antibody while maintaining all other steps to confirm staining specificity. Microscopic evaluation of CCNK expression was conducted using an ECLIPSE E400 microscope (Nikon, Tokyo, Japan), focusing on the intensity and localization of staining. Morphometric analysis was performed at 20× magnification, with three representative areas per sample selected to ensure accuracy and consistency across all evaluated tissue samples. This standardized procedure ensured the reliable and reproducible detection of CCNK expression in the analyzed specimens.

### 2.3. Immunohistochemical Scoring

Immunohistochemical scoring for CCNK expression was conducted using the IRS, which combines the immunointensity score (IS) and immunopercentage score (PS) to provide a semiquantitative evaluation of protein expression levels. Tumor sections were reviewed under a light microscope (ECLIPSE E400, Nikon Instruments Europe, Amsterdam, The Netherlands) at 20× magnification by an experienced pathologist blinded to the clinical and pathological data. For each sample, three randomly selected fields were assessed, and the mean IRS value was calculated. IS represented the staining strength and was graded as follows: 0 = no staining; 1 = weak positive; 2 = moderate positive; and 3 = strong positive. PS reflected the proportion of positively stained cells, categorized as follows: 0 = no positive cells; 1 = 5–24% positive cells; 2 = 25–49% positive cells; 3 = 50–74% positive cells; and 4 = ≥75% positive cells. The final IRS was calculated by multiplying the IS and PS, resulting in values ranging from 0 to 12. High expression of CCNK was defined as IRS ≥ 4, while low expression corresponded to IRS < 4. To dichotomize expression levels, optimal cut-off points were determined using the cut-point function of the Evaluate Cutpoints application in R [22]. This approach allowed for the stratification of samples into “high” and “low” expression groups for subsequent correlation analyses with clinicopathological features and OS. This scoring system provided a standardized and reproducible method for evaluating CCNK expression in tumor tissues.

### 2.4. Database Analysis

TCGA cohort comprised 174 samples diagnosed with endometrial cancer and 23 samples of non-cancerous endometrial tissues, which served as controls. Among the 174 cancer samples, 105 were classified as uterine endometrioid carcinoma, while 69 were identified as non-endometrioid subtypes, including 13 cases of mixed serous and endometrioid carcinoma and 56 cases of serous endometrial adenocarcinoma. RNA sequencing (RNA-seq) transcriptome data were retrieved through the UCSC Xena Browser (http://xena.ucsc.edu/ (accessed on 5 October 2024)) and subsequently normalized using the DESeq2 normalization method. The mRNA expression levels were stratified into high and low groups based on cut-off points identified using Evaluate Cutpoints software, version 4.4.2. Values below 11.25 for *CCNK* were classified as low gene expression, whereas values equal to or exceeding the established cut-off point were considered indicative of high expression. Further analysis was conducted to identify the top 50 genes positively correlated with *CCNK* in uterine corpus endometrial carcinoma (UCEC) utilizing the UALCAN web resource (http://ualcan.path.uab.edu/ (accessed on 2 November 2024)) and TCGA dataset. Pathway enrichment and visualization were performed using the Reactome Pathway Database (https://reactome.org (accessed on 8 November 2024)), while the Kyoto Encyclopedia of Genes and Genomes (KEGG) Pathway Database (https://www.genome.jp/kegg/pathway.html (accessed on 15 November 2024)) was used to explore the pathways associated with the development and progression of endometrial carcinoma. The STRING database (https://string-db.org (accessed on 18 November 2024)) and Cytoscape software, version 3.10.3 with the cytoHubba plugin facilitated the construction of a protein–protein interaction (PPI) network for the top 50 *CCNK*-coexpressed genes. To determine the Gene Ontology (GO) categories—including cellular component (CC), biological process (BP), and molecular function (MF) categories—shared by these genes, the Database for Annotation, Visualization and Integrated Discovery (DAVID; https://david.ncifcrf.gov (accessed on 20 November 2024)) was employed.

### 2.5. Statistical Analysis

All statistical analyses were conducted using GraphPad Prism software (version 7.01, GraphPad Software, La Jolla, CA, USA) and the SPSS Statistics Data Editor (version 26.0, IBM Corporation, Chicago, IL, USA). The Shapiro–Wilk test was applied to assess the normality of the data. Due to the non-normal distribution of the analyzed variables, group comparisons were performed using the Mann–Whitney U test for continuous variables. Fisher’s exact test and the Chi-square test were employed to analyze associations between categorical clinical parameters and the expression of CCNK, classified as a categorical variable. Survival analyses were performed using the Kaplan–Meier method, and comparisons between survival curves were made with the log-rank test. To investigate the effect of CCNK expression on OS, a Cox proportional hazards regression model was used. Univariate analyses were conducted to calculate hazard ratios (HRs) with 95% confidence intervals (95% CIs). Spearman’s rank correlation coefficient was used to assess the relationships between CCNK expression and clinical variables, as well as other biomarkers. Correlation strength was classified according to Guilford’s scale, ranging from no correlation (r = 0) to a full correlation (r = 1). Statistical significance was defined as *p* < 0.05. These methods ensured the comprehensive and rigorous analysis of CCNK expression and its clinical significance.

## 3. Results

### 3.1. CCNK Immunoexpression in Endometrial Cancer and Adjacent Normal Tissue

**Figure 1:**
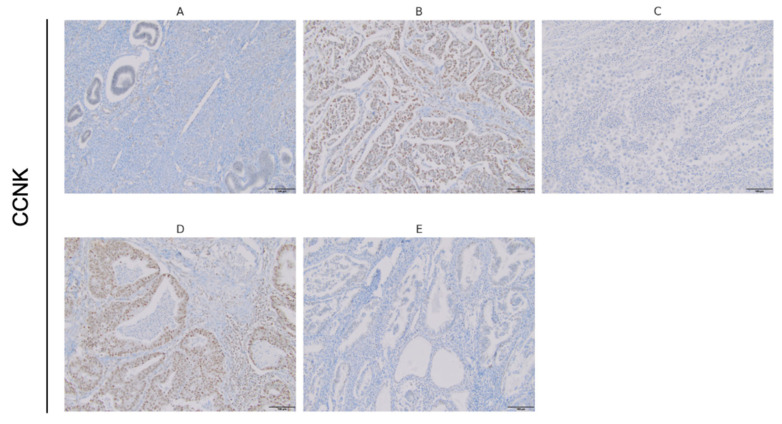
Representative photographs showing immunohistochemical expression of CCNK in EC. Negative control for CCNK, showing no expression (**A**). Serous carcinoma with strong CCNK expression (**B**). Serous carcinoma with low CCNK expression (**C**). Endometrioid carcinoma with strong CCNK expression (**D**). Endometrioid carcinoma with low CCNK expression (**E**). Original magnification: 20×.

No expression of CCNK was detected in normal endometrial tissue, as demonstrated by the negative control, which exhibited an absence of staining (Figure 1A). In serous carcinoma, CCNK expression exhibited significant heterogeneity. Strong nuclear overexpression of CCNK was identified in one case (Figure 1B), while another case showed low nuclear expression (Figure 1C). In both instances of detectable expression, CCNK was localized exclusively within the nucleus. In endometrioid carcinoma, strong nuclear overexpression of CCNK was observed in one sample (Figure 1D), whereas another demonstrated low nuclear expression (Figure 1E).

**Figure 2:**
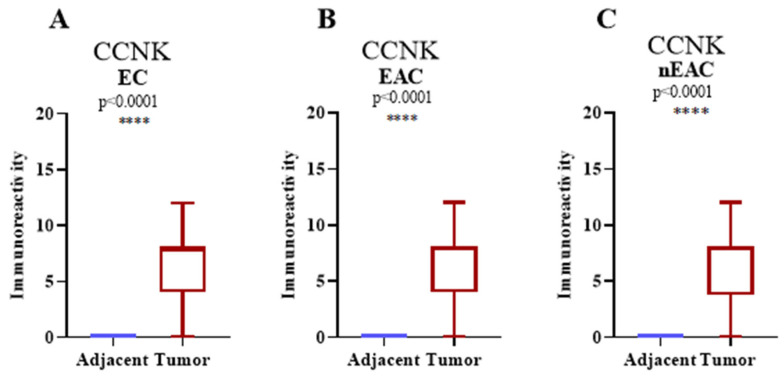
Immunoexpression of CCNK in EC. Immunoexpression of CCNK in histologically normal endometrial tissue compared to all ECs shows significantly higher immunoreactivity in cancerous tissues (**A**). Immunoexpression of CCNK in EAC reveals significantly elevated levels of immunoreactivity in cancerous tissues compared to histologically normal tissue (**B**). Immunoexpression of CCNK in nEAC demonstrates significantly higher immunoreactivity in cancerous tissues compared to histologically normal tissue (**C**). The *X*-axis represents histologically normal endometrial tissue versus cancerous endometrial tissue, and the *Y*-axis displays the IRS. Error bars indicate the maximum and minimum values of the data (**** *p* < 0.0001, Mann–Whitney test).

According to the findings, CCNK immunoreactivity was considerably greater in tumor tissues than in nearby tissues for each of the three endometrial cancer subtypes that was studied: non-endometrioid adenocarcinoma (nEAC), EC, and endometrial adenocarcinoma (EAC). Adjacent tissues displayed incredibly low levels of immunoreactivity for all three comparisons, and for control purposes, their expression levels were set to 0. Highly significant differences between adjacent and tumor tissues were found by statistical analysis using the Mann–Whitney U test (*p* < 0.0001 for all comparisons; Figure 2A–C). This demonstrates how cyclin K is significantly overexpressed in tumor tissues as opposed to nearby non-tumorous tissues.

### 3.2. Tumor Characteristics with Respect to CCNK Immunoexpression

We evaluated whether CCNK reactivity varied based on the clinicopathological features of the patients. When the established definition of low and high expression for each category was applied, 102 (79.69%) patients demonstrated high CCNK expression, while 26 (20.31%) showed low expression. Among patients aged ≤60 years, 32 (78.05%) exhibited high CCNK expression, compared to 70 (80.46%) patients aged >60 years. Regarding histological grade, 6 (66.67%), 61 (83.56%), and 35 (76.09%) patients with G1, G2, and G3 tumors, respectively, demonstrated high CCNK expression. For pT status, high expression was observed in 55 (78.57%), 33 (91.67%), and 14 (70.00%) patients with T1, T2, and T3 + T4 tumors, respectively. Among histological types, 82 (80.39%) endometrioid cancers and 20 (76.92%) non-endometrioid cancers exhibited high CCNK expression. Overall, while high CCNK expression was prevalent across the categories, the analysis showed significant associations between CCNK expression and variables such as pT status (*p* = 0.0499) and FIGO stage (*p* = 0.0433), but no significant associations with age, histologic grade, pN status, pM status, LVSI, or histological type (Fisher’s exact test; Table 1).

### 3.3. Correlation of Overall Survival with CCNK Immunohistochemical Expression

**Figure 3:**
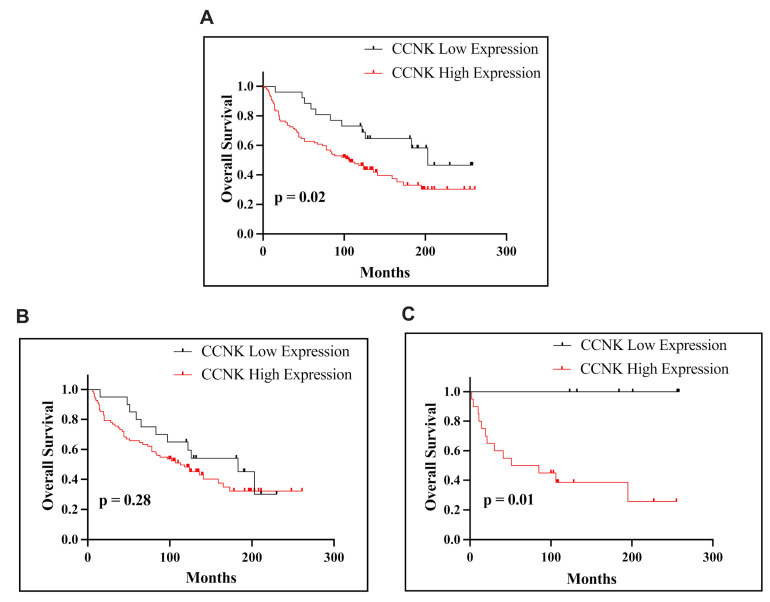
Kaplan–Meier survival curves presenting OS of patients with EC based on CCNK expression. OS according to CCNK immunohistochemical expression in all patients with EC (**A**). OS of patients with endometrioid subtype based on CCNK immunohistochemical expression (**B**). OS of patients with non-endometrioid subtype based on CCNK immunohistochemical expression (**C**). The *p*-values, calculated using the log-rank test, indicate significant differences in survival between groups with low and high CCNK expression.

Kaplan–Meier survival analysis revealed that CCNK immunohistochemical expression, defined using a cut-off value of 4, demonstrated a potential association with OS in patients with endometrial cancer. The median survival time for patients with high and low levels of CCNK expression was 106.5 days. In the overall EC cohort, high CCNK expression was significantly associated with poorer survival times (*p* = 0.02; Figure 3A). This trend was not observed in patients with endometrioid endometrial carcinoma, where no significant differences in OS were detected based on CCNK expression (*p* = 0.28; Figure 3B). However, a significant association was also found in patients with non-endometrioid endometrial carcinoma, where high CCNK expression correlated with poorer survival (*p* = 0.01; Figure 3C).

### 3.4. CCNK mRNA Expression in EC and Normal Tissue Based on TCGA Datasets

**Figure 4:**
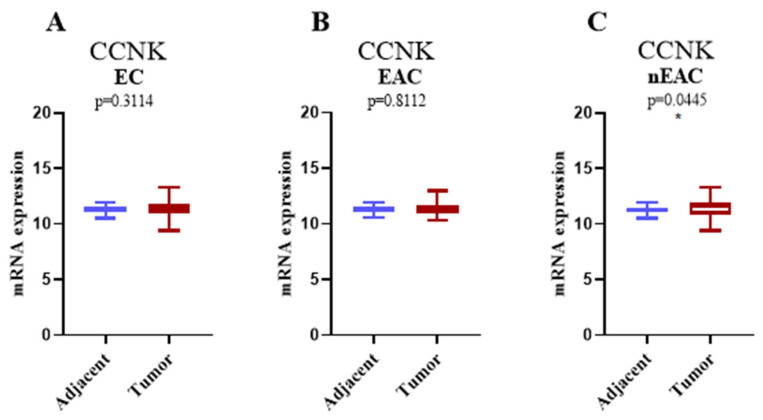
mRNA expression of *CCNK* in EC. mRNA expression of *CCNK* in histologically normal endometrial tissue compared to all ECs shows no significant difference (*p* = 0.3114) (**A**). mRNA expression of *CCNK* in EAC demonstrates no significant difference between histologically normal tissue and cancerous tissue (*p* = 0.8112) (**B**). mRNA expression of *CCNK* in nEAC shows a statistically significant increase in cancerous tissues compared to histologically normal tissue (* *p* = 0.0445) (**C**). The *X*-axis represents histologically normal endometrial tissue versus cancerous endometrial tissue, and the *Y*-axis displays mRNA expression levels. Error bars indicate the maximum and minimum values of the data.

The Mann–Whitney test was used to compare tumor tissues with nearby normal tissues, with Figure 4 displaying the levels of *CCNK* mRNA expression in EC, EAC, and nEAC. There were no discernible variations in *CCNK* mRNA expression in the entire EC cohort (*p* = 0.3114; Figure 4A) or the EAC subtype (*p* = 0.8112; Figure 4B), indicating a consistent expression pattern in both groups. However, in the nEAC subtype, tumor tissues had significantly higher levels of *CCNK* mRNA expression than nearby normal tissues (*p* = 0.0445; Figure 4C), suggesting that *CCNK* may play a part in the development or progression of the more aggressive non-endometrioid subtype of endometrial cancer.

### 3.5. Expression of CCNK-Correlated Genes: In Silico Functional Enrichment Analysis

The top genes positively correlated with *CCNK* were identified using Pearson correlation coefficient and Spearman’s rank correlation. The genes *ALKBH1* and *YY1* showed the highest positive correlation with *CCNK*, with Pearson correlation coefficients of 0.77 and 0.76, respectively. These findings indicate a strong relationship between *CCNK* and these genes, suggesting potential shared regulatory mechanisms or functional roles in the studied context (Table 2).

**Figure 5:**
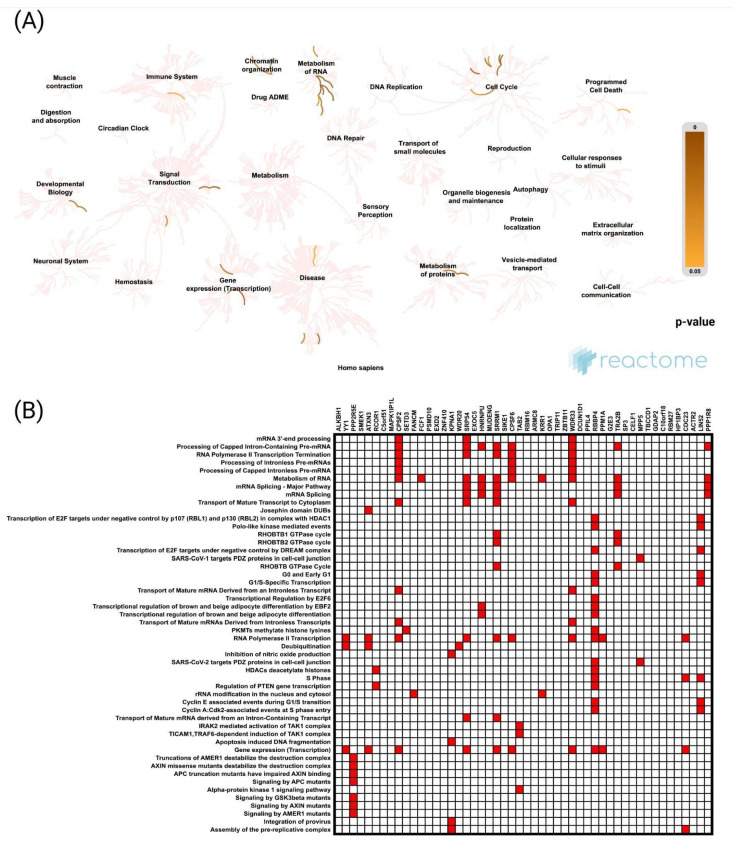
Functional enrichment analysis based on the TCGA dataset and UALCAN web tool. The top 50 genes and reactome pathways positively correlated with *CCNK* expression (**A**,**B**).

Reactome pathway analysis identified significant biological processes associated with genes positively correlated with *CCNK* in UCEC including “Metabolism of RNA”, “Chromatin organization”, and “Gene expression (transcription)”. Additional enriched pathways, such as “Signal transduction” and “Metabolism of proteins”, underscore the involvement of *CCNK*-associated genes in essential cellular mechanisms. Notably, pathways with the most significant enrichment, indicated by darker regions on the map, highlight the pivotal role of *CCNK* in transcriptional regulation, RNA metabolism, and protein processing within cellular functions and disease pathology (Figure 5A).

Further analysis revealed that co-upregulated genes were predominantly enriched in processes such as “mRNA 3′-end processing”, “Processing of capped intron-containing pre-mRNA”, “RNA Polymerase II transcription termination”, “Processing of intronless pre-mRNAs”, and “Processing of capped intronless pre-mRNA” (Figure 5B).

**Figure 6:**
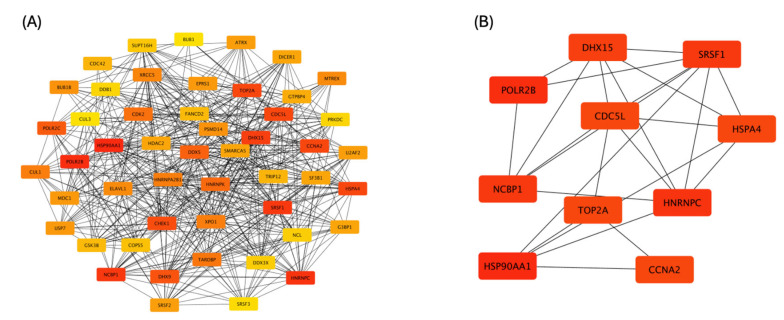
Protein–protein interaction network for genes positively associated with *CCNK*. (**A**) illustrates the interaction network of the top 50 genes exhibiting positive correlation with *CCNK*. Each gene in the network is represented by a node, with its connectivity degree indicated by its color intensity. (**B**) emphasizes the top 10 hub genes within the *CCNK*-correlated network, identified using the CytoHubba plugin in Cytoscape. These hub genes are displayed using a gradient from red to yellow, where a deeper red signifies higher connectivity and greater functional enrichment.

The protein–protein interaction (PPI) networks for genes positively correlated with *CCNK* were constructed using STRING and Cytoscape. The analysis identified 50 nodes connected by 487 edges, indicating strong enrichment (PPI enrichment *p*-value < 1.0 × 10^−16^) with a local clustering coefficient of 0.568. In the PPI network (Figure 6A), genes strongly interacting with *CCNK* were identified, forming a densely connected network. Using the Cytoscape plugin cytoHubba, hub genes were ranked based on their degree of interaction. The top hub genes in this network are highlighted in red, indicating their higher connectivity and importance. Figure 6B focuses on the top-ranked hub genes within the *CCNK*-correlated network, where *HSP90AA1* and *POLR2B* achieved the highest scores. These hub genes may play critical roles in the regulatory pathways associated with *CCNK* and warrant further investigation for their functional relevance in endometrial cancer progression.

**Figure 7:**
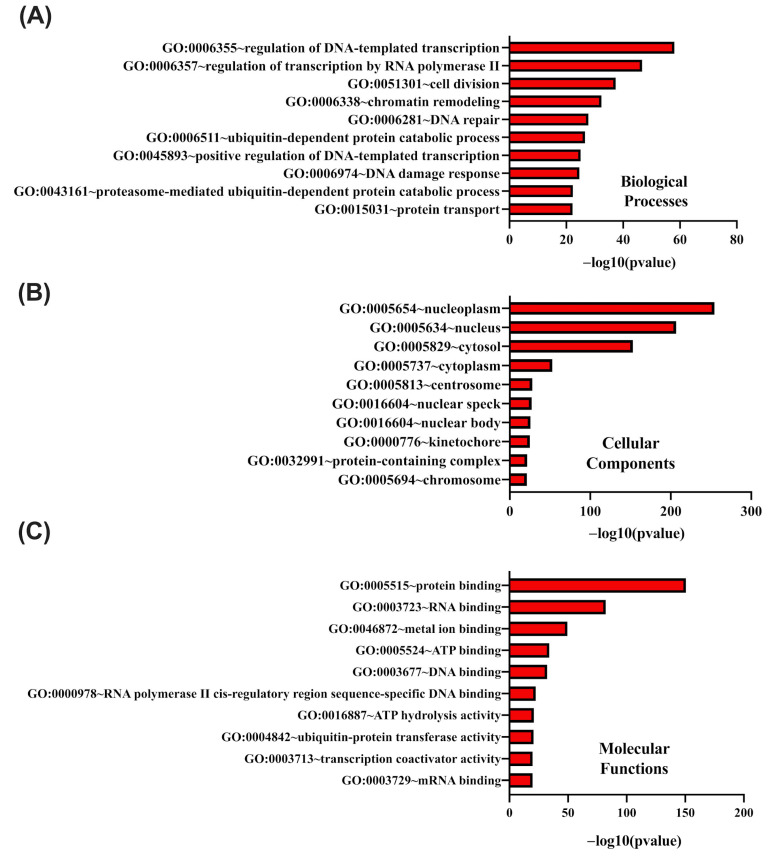
DAVID functional Gene Ontology (GO) analysis of genes co-upregulated with *CCNK*, categorized into BP (**A**), CC (**B**), and MF (**C**). The top 10 GO terms are shown for each category, with *p*-values calculated and ranked based on −log10 (*p*-value).

GO functional enrichment analysis was performed on genes coexpressed with *CCNK*, focusing on their involvement in biological processes, cellular components, and molecular functions. Using the DAVID tool, the analysis identified the most significantly enriched ontology terms in each category. For biological processes, the top enriched terms included GO:0006355 (regulation of DNA-templated transcription), GO:0006357 (regulation of transcription by RNA polymerase II), and GO:0051301 (cell division) (Figure 7A). Additional processes such as chromatin remodeling (GO:0006338), DNA repair (GO:0006281), and protein transport (GO:0015031) were also highly represented. In terms of cellular components, the most enriched terms were GO:0005654 (nucleoplasm), GO:0005634 (nucleus), and GO:0005829 (cytosol) (Figure 7B). Other enriched components included centrosome (GO:0005813), nuclear speck (GO:0016604), and chromosome (GO:0005694). For molecular functions, the top enriched terms included GO:0005515 (protein binding), GO:0003723 (RNA binding), and GO:0046872 (metal ion binding) (Figure 7C). Additional functions such as DNA binding (GO:0003677), RNA polymerase II cis-regulatory region sequence-specific DNA binding (GO:0000978), and ATP hydrolysis activity (GO:0016887) were also prominent. These results highlight the significant roles of *CCNK* and its coexpressed genes in transcription regulation, cellular structural organization, and molecular interactions critical for cellular processes.

### 3.6. Correlation of CCNK mRNA Expression with Survival

**Figure 8:**
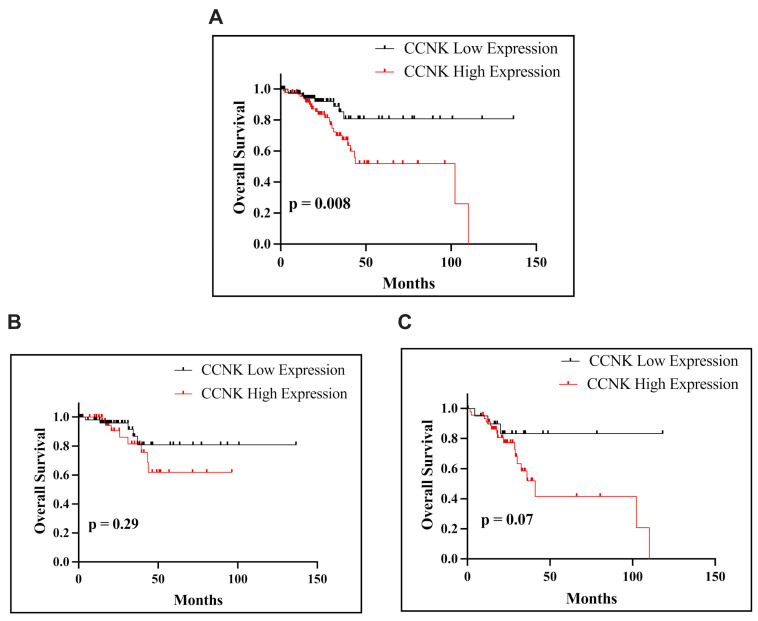
Kaplan–Meier survival curves presenting OS of patients with EC based on *CCNK* expression. OS according to *CCNK* mRNA expression in all patients with EC (**A**). OS of patients with endometrioid subtype based on *CCNK* mRNA expression (**B**). OS of patients with non-endometrioid subtype based on *CCNK* mRNA expression (**C**). The *p*-values, calculated using the log-rank test, indicate significant differences in survival between groups with low and high *CCNK* expression.

The analysis of *CCNK* mRNA expression, using a cut-off value of 11.25, investigated its prognostic relevance for OS in patients with EC. Kaplan–Meier survival analysis demonstrated a statistically significant association between *CCNK* mRNA expression and OS in the overall EC cohort (*p* = 0.008; Figure 8A). However, no significant correlation was observed in patients with endometrioid endometrial carcinoma (*p* = 0.29; Figure 8B) or non-endometrioid endometrial carcinoma (*p* = 0.07; Figure 8C). Nevertheless, across all subgroups, elevated CCNK mRNA expression was consistently associated with a trend toward poorer survival outcomes.

## 4. Discussion

Our study’s findings highlight the possible significance of CCNK as a prognostic biomarker for EC. We found significant correlations between CCNK expression levels and clinicopathological parameters and OS outcomes in patients with EC using a combination of IHC analyses and in silico methodologies that leveraged data from TCGA. These results further support the potential usefulness of CCNK in clinical and therapeutic applications by offering fresh insights into its possible roles in the pathophysiology and progression of EC. To the best of our knowledge, this is the first study evaluating the expression of the CCNK protein in endometrial cancer of the uterine corpus. Given the limited availability of studies on CCNK in endometrial cancer, we compare our findings with data from other cancers, such as lung adenocarcinoma, to gain a more comprehensive understanding of the role of CCNK at both the mRNA and protein levels in tumor progression. The literature data indicate the significant role of cyclin K in the regulation of transcription and control of the cell cycle. Oncogenic activity based on the expression level of the CCNK protein is still poorly understood and requires further research and analysis. Many authors emphasize that cyclin K actively participates in controlling cell proliferation and apoptosis, influencing the development and progression of malignant tumors [23]. To date, the CCNK protein has been studied as a potential biomarker among tumors associated with non-small-cell lung cancer [15,24], the prostate gland [25], the testis [26], and the hematopoietic system [27].

In our study, the nuclear expression of CCNK was observed in endometrial cancer, while no expression was detected in normal endometrial tissue. This is consistent with observations made by other authors who demonstrated that cyclin interacting with CDK9 forms various nuclear complexes and functions as a transcription-regulating factor [28]. In turn, Żuryń et al., in their studies on a non-small-cell lung cancer cell line, demonstrated that the CCNK protein is a newly identified regulator of the Wnt/β-catenin signaling pathway, responsible for the translocation of β-catenin from the nucleus to the cytoplasm, thereby influencing the progression of lung cancer [15,24]. Among studies on cyclin K, the studies by Yao et al., conducted both in vivo and in vitro, deserve special attention. They demonstrated that cyclin K overexpression correlates with poor prognosis in patients with lung cancer. Furthermore, reduced cyclin K expression was shown to decrease proliferation and increase sensitivity to radiation in lung cancer [15]. Lei et al., while analyzing CCNK expression, highlighted its low levels in non-proliferative tissues, which is consistent with the observations in the present study regarding the control group [16]. In turn, Xiang et al. reported the presence of CCNK in normal testicular tissue [26].

Our results revealed that CCNK is significantly overexpressed in EC tissues compared to normal endometrial tissue. Immunohistochemical analysis identified high CCNK expression in 79.69% of EC cases, with a notable correlation with advanced pathological T stage (pT status) and FIGO classification. Interestingly, in both patients with non-endometrioid subtypes and the entire EC cohort, higher CCNK immunohistochemical expression was linked to worse OS. High CCNK expression is associated with poorer survival in all patients with EC and is particularly relevant in the non-endometrioid subtype, where it may contribute to tumor aggressiveness. However, its role in endometrioid EC appears less significant, reflecting the biological and clinical heterogeneity of endometrial cancer. These findings suggest that CCNK’s prognostic impact varies by histological subtype and that it may hold the most promise as a biomarker and therapeutic target in non-endometrioid EC. The findings demonstrated that poor clinical outcomes are associated with high CCNK expression, which is prevalent in endometrial cancer tissues. These results align with earlier research showing that abnormal cyclin expression is frequently linked to the advancement of cancer. Similarly, Yao et al.’s [15] immunohistochemical analysis of lung adenocarcinoma revealed that tumor tissues had significantly higher levels of CCNK protein than nearby normal tissues. Because lower OS was correlated with higher CCNK levels (*p* < 0.05), this overexpression was also linked to a poor prognosis. These similar immunohistochemical results in the two tumors highlight how CCNK promotes tumor growth in an oncogenic manner. The oncogenic role of CCNK is further supported by additional research on breast cancer. In contrast to early-stage lesions like adenosis and fibroadenomas, Lei et al. showed that cyclin K is markedly overexpressed in late-stage invasive ductal and lobular breast carcinomas. One large cohort’s immunohistochemical analyses showed a strong correlation between advanced tumor stages and poor OS and high cyclin K expression. With frequencies ranging from 15% to 40%, cyclin K overexpression was seen in a variety of cancer types, which is consistent with its function in promoting tumorigenesis. Additionally, elevated levels of cyclin K were associated with a lower chance of patient survival [16].

The analysis of *CCNK* mRNA expression in endometrial cancer using the Mann–Whitney test in our study revealed subtype-specific patterns. There were no discernible variations in *CCNK* mRNA expression between tumor tissues and nearby normal tissues in the entire EC cohort (*p* = 0.3114) or the eEAC subtype (*p* = 0.8112), suggesting that these groups exhibited consistent expression patterns. On the other hand, *CCNK* mRNA expression was considerably higher in tumor tissues in nEAC than in nearby normal tissues (*p* = 0.0445). This discovery raises the possibility that *CCNK* has a unique function in the development of nEAC, a more violent form of EC. Furthermore, these outcomes are consistent with our immunohistochemistry data, which showed that higher levels of *CCNK* expression were associated with worse survival outcomes, especially in instances that were non-endometrioid. It is interesting to note that these results are consistent with those of Yao et al., who showed that *CCNK* interacts with important signaling pathways to play a major role in the advancement of cancer. They found that cyclin K interacts with β-catenin to stabilize its protein levels and stimulate the expression of cyclin D1, a gene essential for DNA repair and cell cycle advancement. Cyclin K’s oncogenic significance and importance as a possible biomarker for tumor development and treatment resistance were supported by their investigation, which showed that it is overexpressed in lung cancer tissues and linked to a poor prognosis [15]. Our results broaden our knowledge by suggesting that transcriptional control and cellular homeostasis mechanisms may also be involved in tumor aggressiveness due to elevated *CCNK* expression in nEAC. Although Yao et al. discovered that cyclin K controls the β-catenin/cyclin D1 axis as a significant mechanism in lung cancer, more investigation is needed to ascertain whether the distinct role of *CCNK* in nEAC is due to similar molecular connections. Such research could shed light on the processes behind the aggressive behavior and poor clinical outcomes of this subtype. Another study conducted by Żuryń et al. examined the expression of proteins, including *CCNK*, in NSCLC cells subjected to treatment with sulforaphane (SFN). Using immunofluorescence, flow cytometry, Western blot, and qRT-PCR techniques, the researchers discovered that SFN led to an increase in *CCNK* expression, which was found to be localized within the nucleus. The cell cycle arrest induced by SFN resulted in a decrease in cyclin B1 levels, while levels of cyclin D1 and *CCNK* were found to increase. Elevated levels of CDK12 and CDK13, which form complexes with *CCNK*, were also associated with worse OS outcomes in patients with adenocarcinoma [24].

Our results indicate that the genes most strongly positively correlated with *CCNK* were ALKBH1 and YY1, both of which are involved in transcriptional regulation, chromatin remodeling, and DNA repair. Functional enrichment analysis demonstrates that CCNK and the genes with which it is associated play roles in RNA metabolism, the regulation of transcription by RNA polymerase II, and chromatin organization, among other key biological processes. In order to ensure appropriate gene expression and genomic stability, these results are consistent with the function of cyclin K–CDK12/13 complexes, which phosphorylate the carboxy-terminal domain (CTD) of RNA polymerase II and control transcription elongation and RNA processing [9,29]. Based on these findings, *CCNK* functions as a regulatory hub for maintaining cellular homeostasis, and its dysregulation may encourage carcinogenesis by disrupting these pathways. Pathway analysis revealed enriched biological processes, including mRNA processing, protein transport, and RNA polymerase II transcription termination. These pathways are crucial for maintaining genomic integrity and cellular function, both of which are commonly impaired in cancer. The idea that *CCNK* dysregulation may result in oncogenesis is further supported by cyclin K–CDK12/13 complexes, which are essential for these processes because they facilitate the expression of DNA damage response genes and enhance RNA stability [9,30]. Notably, the analysis of the protein–protein interaction (PPI) network highlighted the significance of HSP90AA1 and POLR2B, verifying the function of *CCNK* in influencing transcriptional activities and aiding tumor development by stabilizing oncogenic transcriptional complexes and regulating essential stress-response pathways for cancer cell survival. Supporting this, other scientific reports identify HSP90 as a promising therapeutic target, underlining its crucial role in preserving the stability of oncogenic proteins and its involvement in the pathways that promote tumorigenesis [31,32].

## 5. Conclusions

Studies suggest that CCNK may play a significant role in the progression and prognosis of EC. Our findings also indicate this, particularly in non-endometrioid subtypes and overall EC cases. Increased CCNK expression is correlated with advanced pathological features and poorer survival outcomes, underlining its potential as a prognostic biomarker and therapeutic target. These findings illustrate the importance of CCNK in key cellular processes such as transcriptional regulation, RNA metabolism, and chromatin organization, indicating its fundamental role in tumor biology. Pathway enrichment and protein interaction analyses further support the idea that CCNK plays a key role as a regulatory hub in these processes. The combination of immunohistochemical data with in silico analyses in this study offers a comprehensive picture of the functional significance of CCNK. Additionally, future research should explore its role in tumor progression and evaluate its potential as a prognostic biomarker, particularly in the context of disease stratification and treatment selection. CCNK expression should also be further examined in the context of its predictive value for treatment response, particularly in patients undergoing chemotherapy or targeted therapies. Identifying its interaction with other molecular markers could help develop combination strategies to improve patient outcomes.

## Figures and Tables

**Table 1:** Characteristics of study population by CCNK immunoexpression group (statistically non-significant results are denoted as “n.s”.).

CCNK				
Variables	Number (%) n = 128	↑n = 102	↓n = 26	*p*-Value
Age				
≤60	41 (32.03)	32 (78.05)	9 (21.95)	n.s
>60	87 (67.97)	70 (80.46)	17 (19.54)	
Histological grade				
G1	9 (7.03)	6 (66.67)	3 (33.33)	n.s
G2	73 (57.03)	61 (83.56)	12 (16.44)	
G3	46 (35.94)	35 (76.09)	11 (23.91)	
pT status				
T1	70 (54.69)	55 (78.57)	15 (21.43)	**0.0499**
T2	36 (28.13)	33 (91.67)	3 (8.33)	
T3	17 (13.28)	10 (58.82)	7 (41.18)	
T4	5 (3.91)	4 (80)	1 (20)	
pN status				
N0	106 (82.81)	84 (79.25)	22 (20.75)	n.s
N1	22 (17)	18 (81.82)	4 (18.18)	
pM status				
M0	116 (90.63)	91 (78.45)	25 (21.55)	n.s
M1	12 (9.38)	11 (91.67)	1 (8.33)	
FIGO				
I	61 (47.66)	49 (80.33)	12 (19.67)	**0.0433**
II	30 (23.44)	27 (90)	3 (10)	
III	26 (20.31)	16 (61.54)	10 (28.46)	
IV	11 (8.59)	10 (90.9)	1 (9.1)	
LVSI				
N	103 (80.47)	80 (77.67)	23 (22.33)	n.s
T	25 (19.53)	22 (88)	3 (12)	
Bokhman histological type				
I	111 (86.72)	89 (80.18)	22 (19.82)	n.s
II	17 (13.28)	13 (76.47)	4 (23.53)	
Histological type				
Endometrioid cancer	102 (79.69)	82 (80.39)	20 (19.61)	n.s
Non-endometrioid cancer	26 (20.31)	20 (76.92)	6 (23.08)	

**Table 2:** Top 50 genes positively correlated with *CCNK* (Pearson coefficient analysis).

*CCNK* (+)-Correlated Gene	PearsonCC	*CCNK* (+)-Correlated Gene	PearsonCC
ALKBH1	0.77	RBM16	0.68
YY1	0.76	ARMC8	0.68
PPP2R5E	0.75	KRR1	0.68
SMEK1	0.74	OPA1	0.68
ATXN3	0.73	TRIP11	0.68
RCOR1	0.73	ZBTB11	0.68
C5orf51	0.71	WDR33	0.68
MAPK1IP1L	0.71	DCUN1D1	0.68
CPSF2	0.71	PPIL4	0.68
SETD3	0.7	RBBP4	0.68
FANCM	0.7	PPM1A	0.68
FCF1	0.7	G2E3	0.68
PSMD10	0.69	TRA2B	0.68
EXD2	0.69	SP3	0.68
ZNF410	0.69	CELF1	0.68
KPNA1	0.69	MPP5	0.68
WDR20	0.69	TBCCD1	0.67
SRP54	0.69	GDAP2	0.67
EXOC5	0.69	C10orf18	0.67
HNRNPU	0.69	RBM27	0.67
MUDENG	0.69	HP1BP3	0.67
SRRM1	0.69	CDC23	0.67
SIKE1	0.69	ACTR2	0.67
CPSF6	0.69	LIN52	0.67
TAB2	0.68	PPP1R8	0.67

## Data Availability

Publicly available datasets were analyzed in this study. These data can be found here: https://ualcan.path.uab.edu/cgi-bin/TCGAExCorrel.pl?genenam=CCNK&cancer=UCEC (accessed on 2 November 2024); https://xenabrowser.net (accessed on 5 October 2024). Our own data presented in this study are available on request from the corresponding author. The data are not publicly available due to ethical restrictions.

## References

[1] Galant N., Krawczyk P., Monist M., Obara A., Gajek Ł., Grenda A., Nicoś M., Kalinka E., Milanowski J. (2024). Molecular Classification of Endometrial Cancer and Its Impact on Therapy Selection. Int. J. Mol. Sci..

[2] Yang Y., Wu S.F., Bao W. (2024). Molecular Subtypes of Endometrial Cancer: Implications for Adjuvant Treatment Strategies. Int. J. Gynecol. Obstet..

[3] Giannone G., Tuninetti V., Ghisoni E., Genta S., Scotto G., Mittica G., Valabrega G. (2019). Role of Cyclin-Dependent Kinase Inhibitors in Endometrial Cancer. Int. J. Mol. Sci..

[4] Bogani G., Monk B.J., Powell M.A., Westin S.N., Slomovitz B., Moore K.N., Eskander R.N., Raspagliesi F., Barretina-Ginesta M.-P., Colombo N. (2024). Adding Immunotherapy to First-Line Treatment of Advanced and Metastatic Endometrial Cancer. Ann. Oncol..

[5] Han K.H., Park N., Lee M., Lee C., Kim H. (2024). The New 2023 FIGO Staging System for Endometrial Cancer: What Is Different from the Previous 2009 FIGO Staging System?. J. Gynecol. Oncol..

[6] Salmon A., Lebeau A., Streel S., Dheur A., Schoenen S., Goffin F., Gonne E., Kridelka F., Kakkos A., Gennigens C. (2024). Locally Advanced and Metastatic Endometrial Cancer: Current and Emerging Therapies. Cancer Treat. Rev..

[7] Goel S., Bergholz J.S., Zhao J.J. (2022). Targeting Cyclin-Dependent Kinases 4 and 6 in Cancer. Nat. Rev. Cancer.

[8] Ding L., Cao J., Lin W., Chen H., Xiong X., Ao H., Yu M., Lin J., Cui Q. (2020). The Roles of Cyclin-Dependent Kinases in Cell-Cycle Progression and Therapeutic Strategies in Human Breast Cancer. Int. J. Mol. Sci..

[9] Xiao Y., Dong J. (2023). Coming of Age: Targeting Cyclin K in Cancers. Cells.

[10] Thomas K.L., Bouguenina H., Miller D.S.J., Sialana F.J., Hayhow T.G., Choudhary J.S., Rossanese O.W., Bellenie B.R. (2024). Degradation by Design: New Cyclin K Degraders from Old CDK Inhibitors. ACS Chem. Biol..

[11] Lv L., Chen P., Cao L., Li Y., Zeng Z., Cui Y., Wu Q., Li J., Wang J.-H., Dong M.-Q. (2020). Discovery of a Molecular Glue Promoting CDK12-DDB1 Interaction to Trigger Cyclin K Degradation. eLife.

[12] Fan Y., Yin W., Hu B., Kline A.D., Zhang V.W., Liang D., Sun Y., Wang L., Tang S., Powis Z. (2018). *De Novo* Mutations of *CCNK* Cause a Syndromic Neurodevelopmental Disorder with Distinctive Facial Dysmorphism. Am. J. Hum. Genet..

[13] Sun R., Wei T., Ding D., Zhang J., Chen S., He H.H., Wang L., Huang H. (2022). CYCLIN K Down-Regulation Induces Androgen Receptor Gene Intronic Polyadenylation, Variant Expression and PARP Inhibitor Vulnerability in Castration-Resistant Prostate Cancer. Proc. Natl. Acad. Sci. USA.

[14] Xiao Y., Dong J., Chen Y. (2023). Abstract 3932: Targeting Cyclin K in Pancreatic Cancer. Cancer Res..

[15] Yao G., Tang J., Yang X., Zhao Y., Zhou R., Meng R., Zhang S., Dong X., Zhang T., Yang K. (2020). Cyclin K Interacts with β-Catenin to Induce Cyclin D1 Expression and Facilitates Tumorigenesis and Radioresistance in Lung Cancer. Theranostics.

[16] Lei T., Zhang P., Zhang X., Xiao X., Zhang J., Qiu T., Dai Q., Zhang Y., Min L., Li Q. (2018). Cyclin K Regulates Prereplicative Complex Assembly to Promote Mammalian Cell Proliferation. Nat. Commun..

[17] Gu X., Shen H., Bai W., Xiang Z., Li X., Zhang R., Shi F., Li H., Zhu G., Guo S. (2022). Endometrial Cancer Prognosis Prediction Using Correlation Models Based on CDK Family Genes. Front. Genet..

[18] Pitolli C., Marini A., Sette C., Pagliarini V. (2024). Physiological and Pathological Roles of the Transcriptional Kinases CDK12 and CDK13 in the Central Nervous System. Cell Death Differ..

[19] Pellarin I., Dall’Acqua A., Favero A., Segatto I., Rossi V., Crestan N., Karimbayli J., Belletti B., Baldassarre G. (2025). Cyclin-Dependent Protein Kinases and Cell Cycle Regulation in Biology and Disease. Signal Transduct. Target. Ther..

[20] Insco M.L., Abraham B.J., Dubbury S.J., Kaltheuner I.H., Dust S., Wu C., Chen K.Y., Liu D., Bellaousov S., Cox A.M. (2023). Oncogenic CDK13 Mutations Impede Nuclear RNA Surveillance. Science.

[21] Houles T., Boucher J., Lavoie G., MacLeod G., Lin S., Angers S., Roux P.P. (2023). The CDK12 Inhibitor SR-4835 Functions as a Molecular Glue That Promotes Cyclin K Degradation in Melanoma. Cell Death Discov..

[22] Ogłuszka M., Orzechowska M., Jędroszka D., Witas P., Bednarek A.K. (2019). Evaluate Cutpoints: Adaptable Continuous Data Distribution System for Determining Survival in Kaplan-Meier Estimator. Comput. Methods Programs Biomed..

[23] Yoshioka H., Noguchi K., Katayama K., Mitsuhashi J., Yamagoe S., Fujimuro M., Sugimoto Y. (2010). Functional Availability of γ-Herpesvirus K-Cyclin Is Regulated by Cellular CDK6 and p16INK4a. Biochem. Biophys. Res. Commun..

[24] Żuryń A., Krajewski A., Klimaszewska-Wiśniewska A., Grzanka A., Grzanka D. (2019). Expression of Cyclin B1, D1 and K in Non-small Cell Lung Cancer H1299 Cells Following Treatment with Sulforaphane. Oncol. Rep..

[25] Schecher S., Walter B., Falkenstein M., Macher-Goeppinger S., Stenzel P., Krümpelmann K., Hadaschik B., Perner S., Kristiansen G., Duensing S. (2017). Cyclin K Dependent Regulation of Aurora B Affects Apoptosis and Proliferation by Induction of Mitotic Catastrophe in Prostate Cancer. Int. J. Cancer.

[26] Xiang X., Deng L., Zhang J., Zhang X., Lei T., Luan G., Yang C., Xiao Z.-X., Li Q., Li Q. (2014). A Distinct Expression Pattern of Cyclin K in Mammalian Testes Suggests a Functional Role in Spermatogenesis. PLoS ONE.

[27] Marsaud V., Tchakarska G., Andrieux G., Liu J.-M., Dembele D., Jost B., Wdzieczak-Bakala J., Renoir J.-M., Sola B. (2010). Cyclin K and Cyclin D1b Are Oncogenic in Myeloma Cells. Mol. Cancer.

[28] Baek K., Brown R.S., Birrane G., Ladias J.A.A. (2007). Crystal Structure of Human Cyclin K, a Positive Regulator of Cyclin-Dependent Kinase 9. J. Mol. Biol..

[29] Edwards M.C., Wong C., Elledge S.J. (1998). Human Cyclin K, a Novel RNA Polymerase II-Associated Cyclin Possessing Both Carboxy-Terminal Domain Kinase and Cdk-Activating Kinase Activity. Mol. Cell. Biol..

[30] Qiu M., Yin Z., Wang H., Lei L., Li C., Cui Y., Dai R., Yang P., Xiang Y., Li Q. (2023). CDK12 and Integrator-PP2A Complex Modulates LEO1 Phosphorylation for Processive Transcription Elongation. Sci. Adv..

[31] Birbo B., Madu E.E., Madu C.O., Jain A., Lu Y. (2021). Role of HSP90 in Cancer. Int. J. Mol. Sci..

[32] Specchia V., Bozzetti M.P. (2021). The Role of HSP90 in Preserving the Integrity of Genomes Against Transposons Is Evolutionarily Conserved. Cells.

